# Supplementary material for: The evolution of constitutively active humoral immune defenses in Drosophila populations under high parasite pressure
Source: PLoS Pathog. 2024 Jan 11;20(1):e1011729. doi: 10.1371/journal.ppat.1011729 (PMC10807768; doi:10.1371/journal.ppat.1011729)
Supplement: S1 Fig — (A) GO enrichment for significantly upregulated genes for populations evolved with high parasitism pressure compared to control populations under uninfected conditions. (B) GO enrichment for significantly upregulated genes for populations evolved with high parasitism pressure compared to control populations after infection by parasitoid wasps. BP–Biological Processes, CC–Cellular Components, MF–Molecular Functions. (PDF) [file ppat.1011729.s001.pdf]

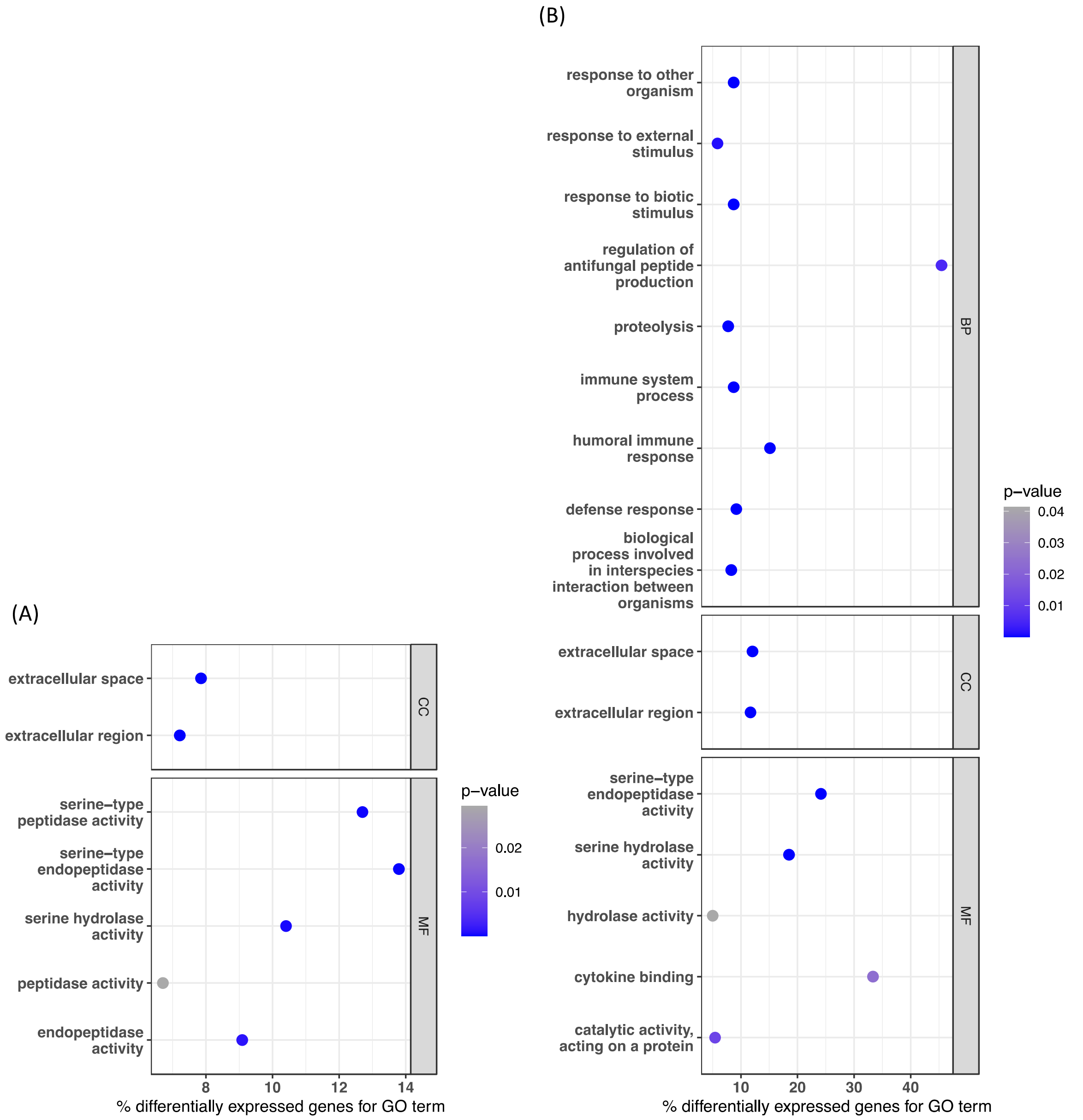

Supplementary Figure 1. Gene ontology (GO) enrichment for significantly upregulated genes comparing between selection regimes. (A) GO enrichment for significantly upregulated genes for populations evolved with high parasitism pressure compared to control populations under uninfected conditions. (B) GO enrichment for significantly upregulated genes for populations evolved with high parasitism pressure compared to control populations after infection by parasitoid wasps. BP – Biological Processes, CC – Cellular Components, MF – Molecular Functions.
